# Maximal safe resection of diffuse lower grade gliomas primarily within central lobe using cortical/subcortical direct electrical stimulation under awake craniotomy

**DOI:** 10.3389/fonc.2023.1089139

**Published:** 2023-02-21

**Authors:** Shujing Yao, Ruixin Yang, Chenggang Du, Che Jiang, Yang Wang, Chongqi Peng, Hongmin Bai

**Affiliations:** Department of Neurosurgery, General Hospital of Southern Theater Command of PLA, Guangzhou, China

**Keywords:** lower-grade glioma, central lobe, awake craniotomy, direct electrical stimulation, maximal safe resection

## Abstract

**Background:**

Diffuse lower-grade glioma (DLGG) in the central lobe is a challenge for safe resection procedures. To improve the extent of resection and reduce the risk of postoperative neurological deficits, we performed an awake craniotomy with cortical-subcortical direct electrical stimulation (DES) mapping for patients with DLGG located primarily within the central lobe. We investigated the outcomes of cortical-subcortical brain mapping using DES in an awake craniotomy for central lobe DLGG resection.

**Methods:**

We performed a retrospective analysis of clinical data of a cohort of consecutively treated patients from February 2017 to August 2021 with diffuse lower-grade gliomas located primarily within the central lobe. All patients underwent awake craniotomy with DES for cortical and subcortical mapping of eloquent brain areas, neuronavigation, and/or ultrasound to identify tumor location. Tumors were removed according to functional boundaries. Maximum safe tumor resection was the surgical objective for all patients.

**Results:**

Thirteen patients underwent 15 awake craniotomies with intraoperative mapping of eloquent cortices and subcortical fibers using DES. Maximum safe tumor resection was achieved according to functional boundaries in all patients. The pre-operative tumor volumes ranged from 4.3 cm^3^ to 137.3 cm^3^ (median 19.2 cm^3^). The mean extent of tumor resection was 94.6%, with eight cases (53.3%) achieving total resection, four (26.7%) subtotal and three (20.0%) partial. The mean tumor residue was 1.2 cm^3^. All patients experienced early postoperative neurological deficits or worsening conditions. Three patients (20.0%) experienced late postoperative neurological deficits at the 3-month follow-up, including one moderate and two mild neurological deficits. None of the patients experienced late onset severe neurological impairments post-operatively. Ten patients with 12 tumor resections (80.0%) had resumed activities of daily living at the 3-month follow-up. Among 14 patients with pre-operative epilepsy, 12 (85.7%) were seizure-free after treatment with antiepileptic drugs 7 days after surgery up to the last follow-up.

**Conclusions:**

DLGG located primarily in the central lobe deemed inoperable can be safely resected using awake craniotomy with intraoperative DES without severe permanent neurological sequelae. Patients experienced an improved quality of life in terms of seizure control.

## 1 Introduction

Diffuse lower grade glioma (DLGG) is one of the most common primary brain tumors, and includes tumors classified as Grade 2–3 astrocytoma by the World Health Organization (WHO), oligodendroglioma, and types with no or slight enhancement on pre-operative magnetic resonance imaging (MRI) studies where postoperative pathology demonstrates focal anaplasia. Conversely, these types of tumors, especially those in or near the eloquent brain areas, are difficult to treat and are often considered inoperable due to their propensity for deep infiltration of the surrounding parenchyma and malignant transformation. However, patients with DLGG achieve long-term survival if they receive early and successful surgical treatment. Furthermore, there has been compelling evidence that an increased extent of resection (EOR) of DLGG could prolong the survival of patients (1–6). Individuals with at least 90% EOR achieved 5-year survival rates of 97%, while patients with less than 90% EOR achieved 5-year survival rates of 76% (1). Therefore, the goal of DLGG surgery should be to maximize the extent of tumor resection while minimizing the risk of postoperative neurological deficits to improve overall survival (OS) and quality of life.

Currently, multiple image-based techniques can be used pre-operatively to help identify eloquent brain areas and define their relationship with brain lesions, including functional magnetic resonance imaging (fMRI), diffusion tensor imaging (DTI), and magnetoencephalography (MEG). Intraoperative aids, such as functional neuronavigation, intraoperative MRI, intraoperative ultrasound, and fluorescent tumor markers, have also been applied to maximize the safety of aggressive resection around eloquent areas. However, these techniques have limited sensitivity and specificity for cortical or subcortical functional mapping (2–5). Consequently, surgeons often face difficulties in distinguishing compensable areas that can be resected and critical areas that must be surgically preserved.

Direct electrical stimulation (DES) mapping is considered the gold standard for neurosurgical planning and can provide a more direct assessment of neuronal function. A recent large meta-analysis that included more than 8000 patients found that glioma resections using intraoperative stimulation mapping were associated with fewer severe late neurologic deficits and more extensive resection, although lesions were more frequent in eloquent areas (6–10).

In this study, we summarized our surgical experience in 15 cases of awake craniotomy with intraoperative DES for DLGG located primarily in the central lobe to explore the efficacy of maximal safe glioma resection using intraoperative cortical and subcortical mapping by DES under awake craniotomy.

## 2 Methods

### 2.1 Patient enrollment

From February 2017 to August 2021, a total of 13 patients (15 operations) suspected of DLGG in the central lobe based on pre-operative MRI were successively treated with awake craniotomy. All the patients recruited had no severe pre-operative neurological deficits or mental disorientation.

Clinical, radiological and histopathological characteristics were collected during admission, including sex, age, symptoms, neurological deficits, Karnofsky performance scale, seizure attacks, tumor location, and pathological grades and subtypes were revised according to the WHO Classification of Tumors of the Central Nervous System, Fifth Edition (CNS 5). The regional ethics committee approved the procedures and all subjects provided their informed written consent prior to participation in this study.

### 2.2 Pre-operative examinations

All patients underwent detailed neurological and psychological evaluations before surgery. Two neurosurgeons completed a neurological function assessment and motor function was scored using a standard muscle strength score ranging from 0 to 5 (0, complete paralysis; 5, entirely normal strength). Neuropsychologists evaluated the general cognitive function of the patients using a brief psychiatric examination. All patients were assessed for handedness using a standardized questionnaire (Edinburgh Handedness Inventory) and were examined with the Mini-Mental State Examination (MMSE). Language function was assessed using an aphasia screening chart, a dysarthria chart, and naming of images. Aphasia screening included oral, written, and sign language comprehension and expression. The dysarthria chart was evaluated by orofacial movement, vowels, and consonant articulation, with a total score of 14 points. The picture naming task was to name 80 black and white pictures with a naming accuracy rate ≥95% being normal. The grade of neurological deficits are presented in **Table 1**. Within 3 days before surgery, MRI was performed using a 3.0-T scanner (GE HealthCare, Chicago, IL, USA) to obtain T1, T2, T2-fluid attenuated inversion recovery (FLAIR), gadolinium enhanced diffuse tensor imaging (DTI), magnetic resonance spectrum (MRS) and perfusion-weighted sequences. All patients were informed in detail about the risks of surgery and the intraoperative stimulation monitoring procedure was performed by a trained nurse responsible for intraoperative motor and language testing.

**Table 1:** Grading of neurological deficits.

Neurological Deficit	Normal	Mild	Moderate	Severe
Muscle strength (Grade)	5	5^-^	4	≤3
Aphasia Screening (correct rate, %)	100	75	50	≤25
Articulation Screening (scores)	14	≥10,<14	≥5,<10	<5
Naming Screening (correct rate, %)	≥95	≥75,<95	≥25,<75	<25

### 2.3 Surgical procedure

As previously described (11), the critical points of awake surgery include patient position, awake anesthesia, neuronavigation, intraoperative ultrasound, DES mapping, and tumor resection. All patients were anesthetized by administration of propofol and remifentanil by target-controlled infusion, using a laryngeal mask airway for intubation during the craniotomy. The ipsilateral critical sensory scalp nerves, pin insertion, and scalp incision sites were injected with local anesthetic (0.67% lidocaine and 0.33% ropivacaine) with 1:200,000 adrenaline to provide rapid and long-lasting local anesthesia while reducing bleeding. Anesthesia was withdrawn to wake up the patient. The location of the tumor was detected intraoperatively using ultrasound before brain mapping and tumor resection. DES mapping was performed using a 5-mm interval bipolar electrical nerve stimulator (Osiris NeuroStimulator; inomed Medizintechnik GmbH, Emmendingen, Germany) with a frequency of 60 Hz, a pulse duration of 1 ms, a current of 2–6 mA (usually 3–4 mA), and a duration of 1 s for motor and sensory tasks and 4 s for language or other cognitive tasks. Positive motor area stimulation was assumed when movements of the contralateral limb or face were induced. Positive stimulation affecting sensory areas was considered when an abnormal feeling was generated in the contralateral limb or face. Positive stimulation of language areas was considered when the patient exhibited counting arrest, anomia, speech repetition, or other language disturbances without twitching of the mouth. After cortical mapping, the lesion was removed by alternating resection and regular subcortical stimulation.

To protect functional pathways, the patient was asked to continue to move their arm and hand or leg, count numbers, or name pictures when the resection moved closer to the subcortical structures. If the patient experienced weakness of the limb, abnormal language, or abnormal sensation, subcortical DES was performed immediately with the same stimulation parameters. If the above-mentioned positive reaction occurred, it was confirmed to be an essential subcortical conduction pathway. The resection was then interrupted in this direction and was continued in other directions. If no positive response occurred, after the patient’s function recovered, resection was continued until the subcortical areas (positive stimulation) or normal meninges (such as the falx cerebri, fissures), ventricles, or arachnoid borders were encountered, or when more than 1 cm outside of normal white matter surrounding the tumor could be visualized. Tumors were resected 2 mm from the sulci near the eloquent brain areas and then were resected inside the pia mater to avoid damage to the vital supplying arteries in the subarachnoid space. Lesions were safely removed to the greatest extent possible to preserve the cortical and subcortical structures of critical functional areas, drainage veins, and supplying arteries.

### 2.4 Postoperative evaluation

Detailed neurological examinations and cognitive function assessments, such as language, were performed 1 day and 5 days after surgery, at discharge, and 3 months after surgery. Neurological dysfunction within 3 months was defined as the early onset stage and after 3 months was defined as the late onset stage. Neurological dysfunction was defined as mild, moderate, or severe based on the assessments described above, which included muscle strength, aphasia detection, articulation disorder detection, and picture naming. Cranial MRI was completed 48 hours after surgery, T2-weighted images or FLAIR imaging was used as a reference, and tumor volume was calculated using 3D Slicer software (v4.6; http://www.slicer.org) (12). Total resection was defined as 100% resection, with 90–100% resection and residual tumor volume < 10 cm^3^ as subtotal resection and < 90% resection and residual tumor volume ≥ 10 cm^3^ considered partial resection.

## 3 Results

### 3.1 Demographic, clinical, and tumor characteristics

From February 2017 to August 2021, a total of 13 patients (15 cases) met the inclusion criteria for this study, including two patients who underwent awake surgery twice in our hospital due to tumor recurrence 3 to 4 years after the first surgery. The patient sample included 7 men and 8 women, aged 24–62 years (average 36.3 years). Twelve patients had experienced seizures before admission, and the other patient also experienced seizures before the second operation. Two patients presented headaches. All patients had MMSE scores ≥ 28. Twelve patients were right-handed and only one patient was ambidextrous.

Pre-operative tumor volumes ranged from 4.3 to 137.3 cm^3^, with a median of 19.2 cm^3^. In all cases, the tumor was primarily in the central lobe (including precentral and postcentral gyri and paracentral lobule) or invaded the central lobe (66.7% of the cases on the left side, 33.3% on the right side). There was involvement of the frontal lobe in nine cases, involvement of the frontoparietal and parietal lobe in two cases and involvement of the insular lobe and parieto-occipital lobe in one case. The exact gyri invaded by the tumors are shown in **Table 2**.

**Table 2:** Demographic data, clinical features, intraoperative mapping, extent of resection, postoperative deficits, and oncological features.

Case N.	Age (year)/sex	Pre-operative symptoms	Lesion location (side)	Lesion volume (cm^3^)	Residual lesion volume (cm^3^)/EOR (%)	Pathology (WHO Grade)		
1	62/F	Seizure	Posterior central gyrus and superior parietal lobule (L)	44.1	4.3/90.2	Astrocytoma (2)/IDH(+)		
2	53/F	Seizure	Posterior central gyrus and superior parietal lobule (L)	16.8	2.4/85.7	Oligoastrocytoma (3)/IDH(+), 1p19q LOH		
3	28/M	Headache	Middle part of the precentral gyrus (L)	4.3	0/100	Astrocytoma (2)/IDH(+)		
4	37/F	Seizure	Superior part of the precentral gyrus and posterior central gyrus (R)	19.2	3.7/80.8	Astrocytoma (2)/IDH(+)		
5	25/M	Seizure	Inferior precentral gyrus and posterior inferior frontal gyrus (R)	36.2	0/100	Astrocytoma (2)/IDH(+)		
6	28/F	Seizure	Inferior precentral gyrus and posterior of inferior frontal gyrus (L)	26.9	0/100	Oligoastrocytoma (2)/IDH(+), 1p19q LOH		
7	28/F	Headache, seizure	Posterior central gyrus and superior and inferior parietal lobule (L)	118.9	0/100	Oligoastrocytoma (2)/IDH(+), 1p19q LOH		
8	54/M	Seizure	Posterior central gyrus and superior parietal lobule (R)	137.3	0/100	Oligoastrocytoma (2)/IDH(+), 1p19q LOH		
9	27/F	Seizure,	Inferior precentral gyrus (L)	13.0	0/100	Oligoastrocytoma (3)/IDH(+), 1p19q LOH		
10/4	40/F	Seizure	Superior precentral gyrus and posterior central gyrus (R)	12.8	1.2/90.6	Astrocytoma (2)/IDH(+)		
11	38/M	Seizure	Hand knob of the precentral gyrus (R)	16.8	0/100	Astrocytoma (2)/IDH(+)		
12	35/M	Seizure	Inferior precentral gyrus and posterior of inferior frontal gyrus (L)	27.4	0/100	Astrocytoma (3)/IDH (–) NEC		
13	34/F	Seizure	Inferior precentral gyrus and posterior of inferior frontal gyrus (L)	26.2	1.4/94.7	Oligoastrocytoma (3)/IDH(+), 1p19q LOH		
14/3	32/M	Seizure	Middle part of the precentral gyrus (L)	5.9	0.5/91.5	Astrocytoma (2)/IDH(+)		
15	24/M	Seizure	Inferior precentral gyrus and posterior of inferior frontal gyrus (L)	34.7	4.8/86.2	Oligoastrocytoma (2)/IDH(+), 1p19q LOH		

Case n.	Cortical sites for motor (n)	Subcortical site for motor (n)	Cortical sites for sensory (n)	Subcortical sites for Sensory (n)	Cortical sites for language (n)	Subcortical sites for language (n)	Early neurological deficits	Late neurological deficits
1	2	1	2	1	0	0	Severe paralysis	Moderate paralysis
2	3	1	1	0	2	0	Moderate paralysis	Normal
3	2	0	2	0	2	1	Severe paralysis and aphasia	Normal
4	2	1	2	0	0	0	Severe paralysis	Normal
5	2	2	1	0	0	0	Moderate aphasia	Normal
6	0	1	1	0	1	0	Moderate aphasia	Normal
7	2	1	3	2	0	2	Severe paralysis	Normal
8	0	0	1	1	0	0	Moderate paralysis	Normal
9	1	2	3	1	1	0	Moderate aphasia	Normal
10/5	1	2	1	0	0	0	Severe paralysis	Normal
11	1	2	3	0	0	0	Moderate paralysis	Normal
12	0	0	0	0	2	1	Severe paralysis and aphasia	Mild paralysis
13	2	0	2	0	3	0	Severe aphasia	Mild aphasia
14/3	1	1	2	0	3	1	Severe paralysis and aphasia	Normal
15	1	0	0	0	1	1	Severe aphasia	Normal

n, number; F, female; M, male; L, left; R, right; EOR, extent of resection; +, mutation; -, wild-type.

### 3.2 Intraoperative DES mapping, tumor EOR, and pathological diagnoses

An awake craniotomy was successfully performed in all cases. After DES in all cases reached a certain intensity (2.0–4.0 mA, median 3.0 mA), DES-induced movement was observed in 13 cases, including 20 cortical sites in 12 cases and 14 subcortical sites in 10 cases. Stimulation-induced sensation was observed in 13 cases, including 24 cortical sites in 13 cases and five subcortical sites in four cases. Twenty-one sites showed language responses in 9 cases (15 cortical areas in 8 cases and 6 subcortical regions in 5 cases). Pyramidal tracts, suprathalamic radiation, or fibers of white matter related to language were identified surrounding the tumor in all patients. Maximum safe tumor resection was achieved according to functional boundaries in all patients.

The mean extent of tumor resection was 94.6%, with 8 cases (53.3%) achieving total resection, 4 (26.7%) subtotal, and three (20.0%) partial resections. The mean residual tumor volume was 1.2 cm^3^. In terms of pathology, tumor grades and subtypes were revised according to the WHO CNS 5. There were 7 cases of diffuse astrocytoma with IDH mutation (Grade 2), 1 case of diffuse astrocytoma with wild-type IDH (Grade 3, NEC), 4 cases of oligodendroglioma with IDH mutation and 1p19q LOH (Grade 2), and 3 cases of oligodendroglioma with IDH mutation 1p19q LOH (Grade 3).

### 3.3 Complications

During surgery, no adverse events related to awake craniotomy were observed. One patient experienced partial DES-induced seizures during resection, which was controlled by cold normal saline irrigation for about 3 minutes.

All patients experienced early postoperative neurological deficits or worsening of symptoms. Nine cases presented severe neurological deficits and 6 showed moderate deficits. Three patients (20.0%) experienced late postoperative neurological deficits at the 3-month follow-up, including one case of mild Broca’s aphasia and 2 cases of inflexible movements (one mild, one moderate). One patient (6.7%) experienced moderate late neurological sequelae after awake craniotomy for DLGG in the central lobe. None of the patients experienced post-operative late onset severe neurological impairments. Ten patients with 12 tumor resections (80.0%) had resumed normal activities of daily living at the 3-month follow-up.

Among the 14 cases of pre-operative epilepsy, 12 (85.7%) were seizure-free after receiving antiepileptic drugs from 7 days after surgery up to the last follow-up.

### 3.4 Illustrative cases

#### 3.4.1 Case 1

**Figure 1:**
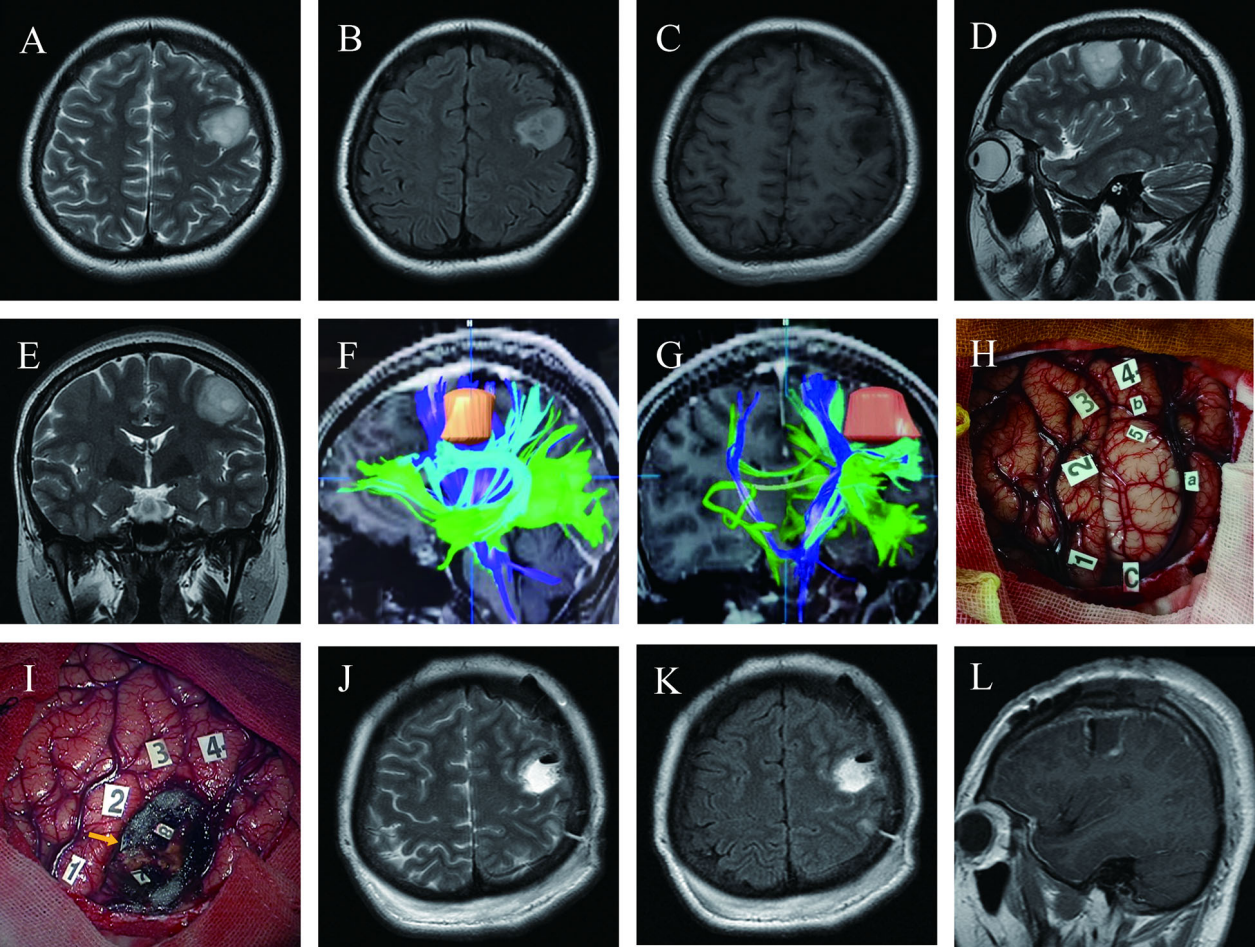
Maximum safe resection of oligodendroglioma harboring IDH mutation and 1P/19Q codeletion (WHO Grade 3) in the inferior left precentral gyrus under awake craniotomy in a 27-year-old woman presenting with seizures (Case 9); **(A–E)** Pre-operative T2 axial, T2 FLAIR axial, T1-weighted gadolinium-enhanced axial, T2 sagittal, and T2 coronal MRI revealed the tumor’s location precisely in the inferior lateral left hand knob of the precentral gyrus. **(F, G)** DTI-based reconstructed fibers showed arcuate fasciculus beneath the lesion and slightly distorted, and the pyramidal tract was medial to the lesion; **(H)** Intraoperative view before tumor resection; Tumor borders marked by letters (a, anterior; b, superior; c, inferior). Number of tags denotes positive DES mappings (1, paresthesia of the right little and ring fingers; 2, numbness of the right thumb; 3, paresthesia of the right corner of mouth; 4, motor responses in the right corner of mouth; tag 5 is on the surface of the tumor, where speech arrest and convulsions of the right corner of the mouth were induced during DES); **(I)** Intraoperative view after tumor resection based on the functional boundary; The tumor was removed until DES mapping encountered eloquent brain areas at cortical and subcortical levels (Tag 7, movement of the right hand during DES; 8, movement of the right corner of the mouth; 9 with a yellow arrow, an electrifying sensation of the right index finger); **(J–L)** Twenty-four hours postoperative T2 axial, T2 FLAIR axial, and T1-weighted gadolinium-enhanced sagittal MRI demonstrated total tumor resection.

A 27-year-old right-handed woman (case 9) presented a two-week history of transient language disruptions accompanied by loss of consciousness. Physical examination revealed no neurological impairments, and pre-operative MRI revealed a low-grade glioma in the left central lobe (**Figures 1A–E**). DTI-based fiber tracking showed that the arcuate fasciculus and the pyramidal tract were below and medial to the lesion, respectively (**Figures 1F, G**). After revealing the dura, intraoperative ultrasound showed a tumor in the inferior part of the left precentral gyrus. Eloquent cortices, including sensory, motor, and language areas, were found by cortical mapping (**Figure 1H**). Three subcortical sensory and motor sites were detected during tumor resection using DES (**Figure 1I**). Postoperative MRI revealed total tumor resection (**Figures 1J–L**). The patient experienced moderate aphasia about one week after surgery, with normal language function at discharge.

#### 3.4.2 Case 2

**Figure 2:**
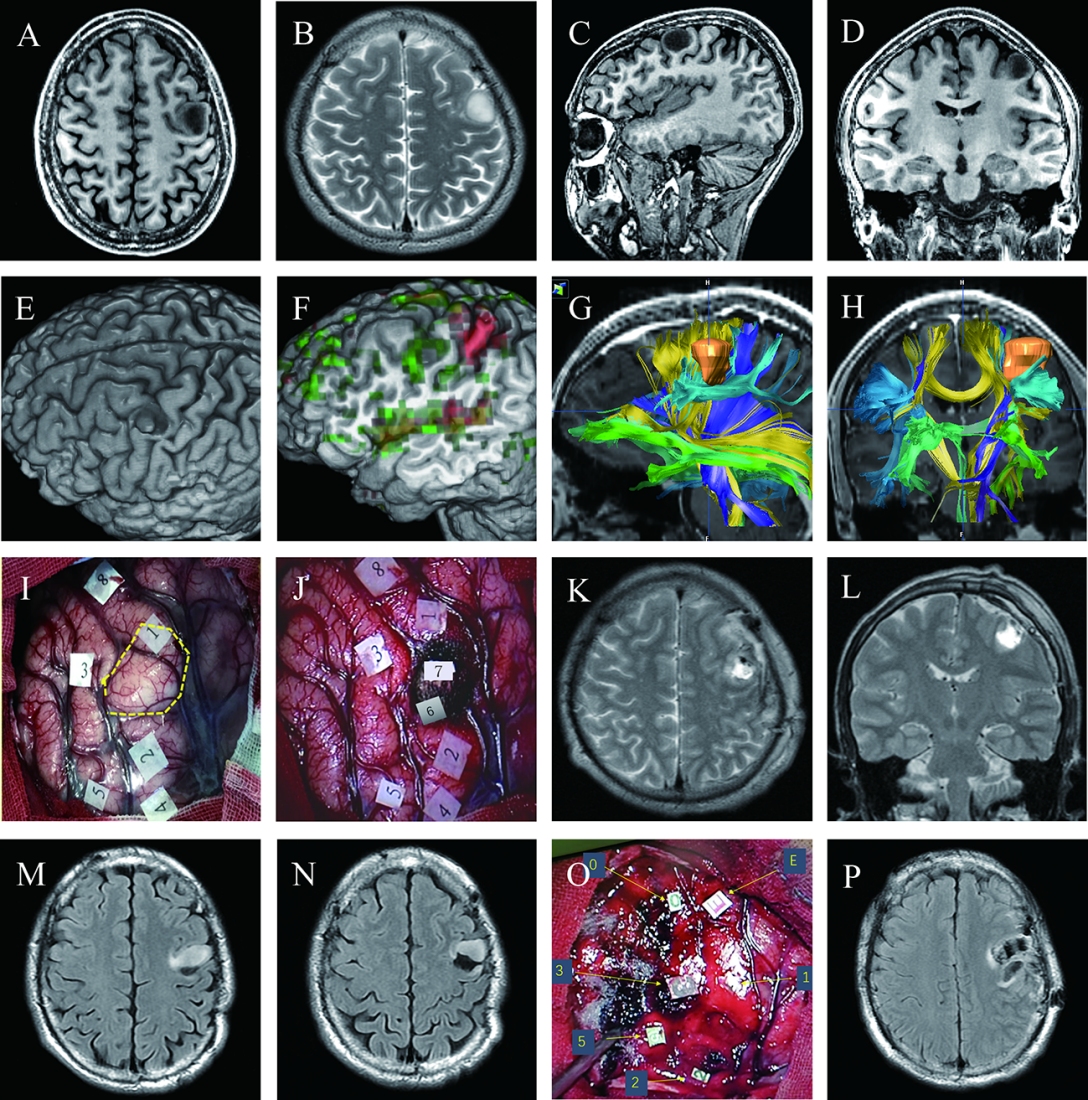
Maximum safe resection of astrocytoma with IDH mutation (WHO Grade 2) in the inferior left precentral gyrus in two successive awake craniotomies over four years (Cases 3 and 14); **(A–D)** Pre-operative T1 axial, T2 axial, T1 sagittal, and T1 coronal MRI; **(E)** Surface rendering of pre-operative T1-weighted MRI revealed a tumor in the middle of the left precentral gyrus, which was separated into the dorsal and ventral parts by the tumor; **(F)** Surface rendering of functional areas overlapped with T1-weighted MRI and fMRI; Red denotes active sites for hand grasp task; green represents active sites for naming task; **(G, H)** Pre-operative planning using neuro-navigation showed a close relationship between the tumor and white matter fibers; **(I)** Intraoperative view before tumor resection; An ultrasonic tumor border is marked with the dotted yellow circle. Numbers on tags denote zones of positive DES mapping (2 and 4, finger movement; 3, mouth sensation; 5, finger sensation; 1, speech arrest during counting; 8, anomia during naming)**(J)** Intraoperative view after tumor resection; glioma was removed until eloquent neural structures were encountered at cortical and subcortical levels using subcortical DES; Tag 6, pyramidal tract for right thumb movement; 7, area responsible for speech arrest during counting; Postoperative axial **(K)** and coronal **(L)** T2 FLAIR-weighted MRI demonstrated total tumor resection; **(M, N)** Follow-up MRI revealed a recurrent tumor anterior to the previous surgical residual cavity; **(O)** Intraoperative view of the second surgery before tumor resection; Number and letter tags denote zones of positive DES mapping (2, thumb movement; 3, mouth sensation; 5, finger sensation; 0, 1, and E, both speech arrest and interrupt of hand grasp during a dual coordinate task); **(P)** Postoperative axial T2 FLAIR MRI showed subtotal tumor resection with a residue of 0.5 cm^3^.

A 28-year-old right-handed man (case 3) presented with a headache. A surface rendering of pre-operative T1-weighted MRI revealed a tumor located entirely within the precentral gyrus (**Figures 2A–E**). The surface rendering of the functional areas overlapped with T1-weighted MRI and fMRI identified the active sites for hand grasping and naming tasks (**Figure 2F**). DTI-based fiber tracking showed a close relationship between the tumor and white matter fibers (**Figures 2G, H**). Under awake craniotomy using cortical and subcortical DES, the maximum safe tumor resection was achieved with an EOR of 100% (**Figures 2I–L**). The patient experienced paralysis of the right hand and Broca aphasia three days after surgery, but resumed normal life 3 months after surgery. The histological diagnosis was astrocytoma with IDH mutation (Grade 2). This patient presented seizures 40 months after surgery, with MRI revealing a recurrent tumor anterior to the residual surgical cavity (**Figures 2M, N**). Thus, we performed a second awake craniotomy (case 14), with subtotal tumor resection and an EOR of 91.5% (**Figures 2O, P**). Following surgery, the patient experienced severe paralysis and aphasia, but recovered to normal at the 3-month follow-up.

#### 3.4.3 Case 3

**Figure 3:**
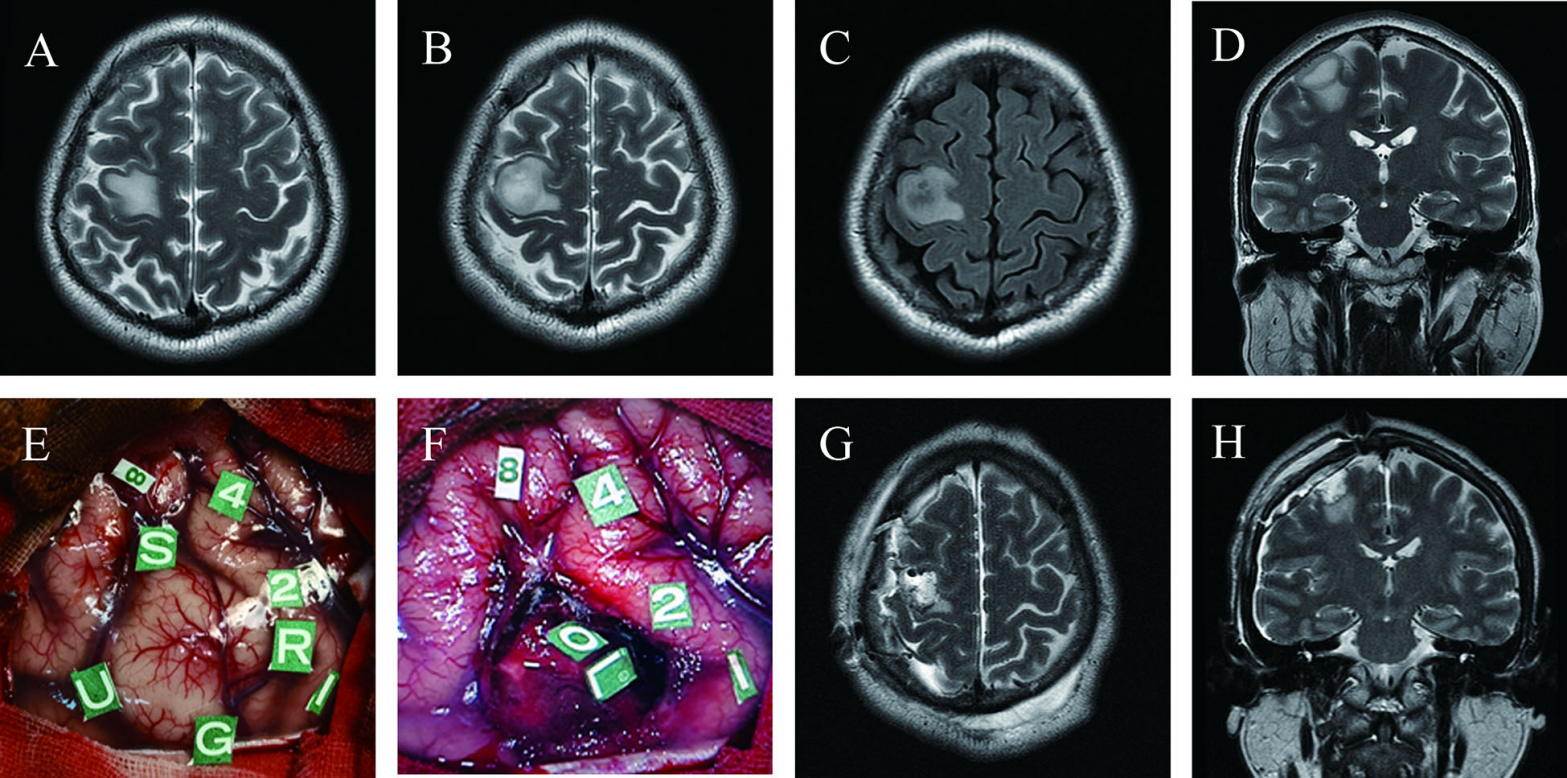
Maximum safe resection of astrocytoma with IDH mutation (WHO Grade 3) in the hand knob of the right precentral gyrus under awake craniotomy in a 38-year-old man presenting with seizures (Case 11); Pre-operative T2 axial **(A, B)**, T2 FLAIR axial **(C)** and T2 coronal **(D)** MRI revealed the tumor’s location precisely on the hand knob of the right precentral gyrus; **(E)** Intraoperative view before tumor resection; Tumor borders marked with letter tags (Tag U, anterior; G, superior; S, inferior; R, posterior). Number tags show positive points of DES mapping (8, changes in vocal tone; 1, 2, 4, primary sensory cortex, paresthesia of the left forearm, palm, and thumb, respectively); **(F)** Intraoperative view subsequent to tumor removal; Tag L, subcortical area for wrist movement; O, subcortical area for finger movement; Postoperative axial **(G)** and coronal **(H)** T2-weighted MRI demonstrated total tumor resection.

A 38-year-old man (case 11) experienced recurrent left hand convulsions one month before admission. MRI revealed a low-grade glioma located precisely in the hand knob of the right precentral gyrus (**Figures 3A–D**). The pre-operative physical examination showed normal muscle strength in all limbs. To achieve maximum safe tumor resection, the patient underwent awake craniotomy (**Figures 3E, F**), resulting in total tumor resection with cortical and subcortical boundaries (**Figures 3G, H**) and moderate transient postoperative paralysis at one week after surgery.

## 4 Discussion

DLGG frequently occurs in young patients and life expectancy is longer in patients with active social and professional lives (1, 13). The first-line therapeutic option for DLGG is maximum safe resection, with EOR being a significant independent prognostic factor. Studies have shown that higher EOR is associated with better progression-free survival and OS (1–6, 14–18). Even some cases with grade 3 or 4 transformation foci with a grade 2 background do not necessarily require immediate adjuvant therapy following a radical maximal safe resection under awake craniotomy. Therefore, we selected patients with DLGG having a grade 2 background, which included four cases presenting focal grade 3 anaplasia as our cohort (19). Furthermore, because oligodendroglioma is sensitive to radiotherapy and chemotherapy, some clinicians have a conservative attitude toward surgical resection. When oligodendroglioma is located in eloquent brain areas, to avoid postoperative neurological deficits, some clinicians sustain that excessive resection is not necessary, and elect only partial resection or biopsy as the therapeutic option. It is challenging to classify tumors as oligodendroglioma or astrocytoma pre-operatively. Furthermore, recent research has also confirmed that the EOR of oligodendroglioma is closely related to prognosis. Studies registered in the extensive Surveillance, Epidemiology and End Results database and in the National Cancer Database revealed that the extent of resection was associated with an increase in OS for both histologically confirmed oligodendrogliomas and molecularly defined tumors (IDH mutations with 1p/19q-codeletion) (17, 20). However, the major challenge in neurosurgery is to eradicate the tumor as much as possible while maximally preserving neurological functions. Various techniques have been introduced to achieve this goal, such as fluorescence-guided surgery, intraoperative ultrasound, intraoperative MRI combined with functional neuronavigation, and Raman spectroscopy (21–27).

Although fMRI, a noninvasive mapping method, is becoming increasingly applied in neurosurgery, its precision remains controversial and the parameters used in different studies vary significantly (9, 19, 21–23, 28). Recently, Weng et al. (21) performed a systematic review that included ten studies with a total of 214 patients with brain tumors to assess the accuracy of fMRI for language mapping with direct cortical stimulation and found that, per patient, the pooled sensitivity and specificity of fMRI was 44% and 80%, respectively; per tag, the pooled sensitivity and specificity were 67% and 55%, respectively. Another meta-analysis by Metwali et al. (23) included six studies of language activation and two of motor activation. The study concluded that fMRI alone (due to neurovascular uncoupling) or analysis of the findings present limitations in reliability compared to direct cortical stimulation, and using fMRI alone for surgical planning could lead to undesirable outcomes. Additionally, a clinical survey conducted by Stopa et al. on the use and attitudes of neurosurgeons towards fMRI as a surgical planning tool in neurooncology patients revealed that 70% of the responders presented a resected fMRI positive functional site, of which 77% did so because the area was ‘cleared’ using intraoperative cortical stimulation. If the results of fMRI and intraoperative mapping disagreed, 98% of the respondents would rely on intraoperative mapping (25).

DTI tractography, a noninvasive method for visualizing white matter tracts, can provide clinically relevant information during pre-operative planning and intraoperative mapping for brain tumor resection (8, 21, 25). However, DTI tractography relies only on the indirect reconstruction of fibers based on measuring the diffusion of water molecules. The results depend on many factors, including data acquisition, geometrical models, software programs, and regions of interest (7, 9, 10, 23, 26, 27). Maier-Hein reported that most state-of-the-art algorithms produce tractograms containing 90% of the ground-truth bundles, while the same tractograms have many more invalid than valid bundles. Consequently, DTI tractography is not sufficiently reliable to be the basis for neurosurgical decision making, and the possibility of incorrectly displayed fibers leads to a risk of postoperative deficits for the patient (2).

It should be mentioned that to date, intraoperative DES mapping under awake anesthesia remains the standard goal for brain surgery, especially at the subcortical level (11, 12, 28–37). In the present study, we performed an awake craniotomy in patients pre-operatively suspected of DLGG located primarily in the central lobe. The postoperative outcomes also illustrated the power of this procedure to detect functional tissue around tumors. From a traditional standpoint, DLGG is considered inoperable in the central lobe, which is composed of the pre- and postcentral gyri and the paracentral lobule, which are the eloquent brain areas (38–41). According to our experience in this study, awake surgery and DES can also achieve maximum safe resection of DLGG when it is located entirely in the central lobe due to functional remodeling of the brain.

In this retrospective report, functional white matter fibers were identified surrounding the tumor in all patients, and maximum safe tumor resection was achieved according to functional limits in all patients. The mean extent of tumor resection was 94.6%. The mean tumor residue was 1.2 cm^3^. Only one patient (6.7%) experienced moderate late onset postoperative neurological deficits and none of the patients experienced severe late neurological impairments. Among 14 cases with pre-operative epilepsy, 12 patients (85.7%) were seizure-free after taking antiepileptic drugs starting 7 days post-operatively to the last follow-up after surgery (3 months). Lower-grade glioma located primarily in the central lobe can be safely resected using awake craniotomy with intraoperative DES without severe permanent neurological sequelae. All patients achieved a better quality of life with respect to seizure control.

Although the application of subcortical DES to remove DLGG in functional areas can reduce the incidence of late onset neurological dysfunction, the incidence of early neurological dysfunction is high. All the patients in the present cohort exhibited early neurological dysfunction, which may be related to postoperative tumor cavity edema, ischemia, or damage to some auxiliary functions of the cortical and subcortical fibers. However, early postoperative neurological dysfunction will prolong hospital stays, and most patients require rehabilitation treatment, leading to increased medical costs.

During awake craniotomy procedures, surgeons should pay closer attention to the following details: 1) maintain the integrity of the fiber tracts of white matter as subcortical electrical stimulation is as essential as cortical mapping, and the tumor should be excised using alternating resection and regular subcortical stimulation (42–44); 2) ensure postoperative arterial supply and venous drainage of surrounding normal brain tissue, and some vital blood vessels must be preserved, such as the central sulcus artery, the artery of pre- and post-central sulcus artery, the paracentral artery, and veins of Labbé and Trolard (41, 42); 3) the functional boundaries detected by intraoperative DES and pia mater can be used as essential protective tissue to avoid injury to important blood supply arteries located in the cerebral sulcus.

There are some limitations to this study. First, this was a single-center retrospective study with a small sample size, and patient selection was based on the economic status and intraoperative cooperation of awake craniotomy of the patient. Therefore, to evaluate the fundamental role of awake craniotomy surgery for DLGG in the central lobe, further prospective and randomized multicenter cohort studies with larger sample sizes are required. Second, in such a clinical study, some mixed factors could lead to potentially biased results. To reduce bias in surgical procedures, surgeries in all cases were performed by the same team composed of experienced neurosurgeons, anesthetists, and trained nurses. Third, we attempted to analyze the relationship between seizure control after surgery and LGG EOR. However, no definitive conclusion could be drawn due to the small sample size, although the incidence of seizure after tumor total resection was seemingly lower than that of nontotal resection.

## 5 Conclusions

Based on our experience in this study, DLGG located exclusively in the central lobe and considered inoperable can be safely resected with a mean EOR of nearly 95% under awake anesthesia with intraoperative DES. Although numerous non-invasive imaging techniques are becoming increasingly popular and accurate, their validity in identifying eloquent cortical areas and white matter tracts is still inferior to intraoperative DES. However, a prospective and more extensive randomized cohort studies are needed to evaluate the fundamental role of awake craniotomy surgery for DLGG in the central lobe.

## Data availability statement

The raw data supporting the conclusions of this article will be made available by the authors, without undue reservation.

## Ethics statement

The studies involving human participants were reviewed and approved by the ethics committee of the General Hospital of Southern Theater Command of PLA. The patients/participants provided their written informed consent to participate in this study. Written informed consent was obtained from the individual(s) for the publication of any potentially identifiable images or data included in this article.

## Author contributions

SY, RY, and CP participated in data collection and analysis, and writing of the paper. CJ and YW participated in the pre- and post-operative evaluation. HB participated in brain mapping and data interpretation. HB and CD reviewed and edited the manuscript. All authors contributed to the article and approved the submitted version.
